# *miR-34b/c* rs4938723 T>C Decreases Neuroblastoma Risk: A Replication Study in the Hunan Children

**DOI:** 10.1155/2019/6514608

**Published:** 2019-09-10

**Authors:** Yong Li, Zhen-Jian Zhuo, Haiyan Zhou, Jiabin Liu, Zhenghui Xiao, Yaling Xiao, Jing He, Zan Liu

**Affiliations:** ^1^Department of Pediatric Surgery, Hunan Children's Hospital, Changsha, 410004 Hunan, China; ^2^Department of Pediatric Surgery, Guangzhou Institute of Pediatrics, Guangzhou Women and Children's Medical Center, Guangzhou Medical University, Guangzhou, 510623 Guangdong, China; ^3^Department of Pathology, Xiang-ya School of Medicine, Central South University, Changsha, 410013 Hunan, China; ^4^Emergency Center of Hunan Children's Hospital, Changsha, 410004 Hunan, China

## Abstract

Neuroblastoma is the most common seen solid neural tumor in children less than age one. As mutation in the *miR-34b/c* gene is observed in several types of human malignancies, there likely to be similar events that contribute to the pathogenesis of neuroblastoma. We hypothesize that polymorphism in the *miR-34b/c* gene might predispose to neuroblastoma. Here, we conducted this replication study by genotyping rs4938723 T>C from *miR-34b/c* in Hunan children (162 subjects with neuroblastoma and 270 control subjects) and examined its effect on the risk of neuroblastoma. We determined such association using logistic regression, adjusted for age and gender. Relative to those with TT genotype, subjects with C allele had reduced neuroblastoma risk (TC vs. TT: adjusted OR = 0.46, 95%CI = 0.30‐0.71; additive model: adjusted OR = 0.64, 95%CI = 0.47‐0.88; TC/CC vs. TT: adjusted OR = 0.49, 95%CI = 0.33‐0.73). Stratified analysis revealed that rs4938723 TC/CC carriers were less likely to develop neuroblastoma for patients in the subgroups of age ≤ 18 months, age > 18 months, females, males, tumors in retroperitoneal, tumors in other sites, and clinical stages II, III, IV, and III+IV. Our findings verified *miR-34b/c* rs4938723 C variant allele as a protective factor for the risk of neuroblastoma. Further investigation of how *miR-34b/c* rs4938723 T>C might modify neuroblastoma risk is warranted.

## 1. Introduction

Neuroblastoma is a childhood tumor that mainly derives from neural crest progenitor cells [1–3]. Despite representing about 8-10% of all pediatric cancer diagnoses, neuroblastoma disproportionately results in 12-15% of all childhood cancer-related mortality [4–6]. It is characterized by widely clinical heterogeneity, spans from spontaneous regression to therapy-refractory progression [7]. Another reflection of such heterogeneity was the contrasting survival rate of different subgroup patients [8, 9]. In patients with the low- and intermediate-risk neuroblastoma, the long-term survival rate is greater than 90% [10]. However, in patients with the high-risk neuroblastoma, less than 40% could finally survive [11, 12].

In the past decades, considerable progress has been made in understanding the genetic underpinnings of neuroblastoma. Exposed environmental factors of children and pregnant women were reported to predispose to neuroblastoma, but not finally defined [13, 14]. Mutations in *ALK* [15] and *PHOX2B* [16] were considered as two major causes of familial neuroblastoma. Other SNPs in genes including *LMO1* [17], *BARD1* [18], *TP53* [19], *LIN28B* [20], *HACE1* [20], *NEFL* [21], and *CDKN1B* [22] have more recently been identified to be associated with neuroblastoma predisposition. Moreover, the association of these SNPs to neuroblastoma risk has also been replicated in many other populations, especially the SNPs in the *BARD1* gene [23–25]. Taken together, however, all the current identified mutations still could not fully elucidate the etiology of neuroblastoma. We are still on the way to fully reveal the genetic landscape of neuroblastoma. Identification of other somatic mutations will further clarify the mechanisms of neuroblastoma.

MicroRNAs (miRNAs) are a class of nonprotein-coding, small, single-stranded RNAs with about 22 nucleotides [26]. miRNAs participate in transcriptional regulation through multiple mechanisms, including mRNA degradation, translational repression, or cleavage of mRNA [26–28]. In the past decade, more and more miRNAs are being identified that play vital regulatory roles in human disorders, including cancers. Mutations or single nucleotide polymorphisms (SNPs) in miRNA genes may alter the binding ability of miRNAs to their target mRNAs, thus resulting in diverse functional consequences and thereby possibly impact cancer susceptibility [29, 30]. rs4938723 T>C is located at the promoter region of *pri-miR-34b/c* [31]. Such T to C shift polymorphism might cause a disruption of GATA-X transcription factor binding capacity, which results in decreased *pri-miR-34b/c* expression [32]. Thus far, most studies have addressed the identification of *miR-34b/c* rs4938723 T>C in breast cancer [33], colorectal cancer [34], hepatocellular cancer [35], and nasopharyngeal carcinoma [36], whereas few studies focused on the role of *miR-34b/c* gene rs4938723 T>C in neuroblastoma risk. In our previous study conducted recently, we firstly found that rs4938723 T>C polymorphism was associated with a significantly decreased neuroblastoma risk [37]. Here, we further conducted a replication hospital-based case-control study aiming to verify the association between *miR-34b/c* rs4938723 T>C and neuroblastoma risk in Hunan children.

## 2. Materials and Methods

### 2.1. Study Subjects

Prior to analysis, the study protocols were approved by the Institutional Review Board of Hunan Children's Hospital. The current case-control study was carried out in Hunan Children's Hospital. A total of 162 cases were pathology-confirmed with neuroblastoma, and 270 controls with no prior history of neuroblastoma were randomly enrolled in the same area as cases. All guardians of participants provided written informed consent. The detailed information of selection criteria of study subjects was reported in our previous paper [38–40].

### 2.2. Genotyping

Genomic DNA was isolated from venous blood using a TIANamp Blood DNA Kit (TianGen Biotech Co. Ltd., Beijing, China). Genotype analysis of *miR-34b/c* gene rs4938723 T>C was undertaken using TaqMan SNP genotyping assay from Applied Biosystems [41–44]. Negative controls (with water) and duplicate test samples (10% of all the samples) were included in each 384-well plate. 100% concordant of genotypes in replicates were achieved.

### 2.3. Statistical Analysis

Tests for the Hardy-Weinberg equilibrium (HWE) were conducted for *miR-34b/c* rs4938723 T>C among control subjects with the use of the *χ*^2^ test. Differences in demographic variables between case subjects and control subjects were analyzed using the two-sided *χ*^2^ test. Neuroblastoma risk was determined as odds ratios (ORs) and 95% confidence intervals (CIs), based on unconditional logistic regression adjusted for age and gender. A *P* value of <0.05 was used for statistical significance. The SAS release 9.1 (SAS Institute, Cary, NC) was used for statistical analyses.

## 3. Results

### 3.1. Association between miR-34b/c rs4938723 T>C and Neuroblastoma Susceptibility

A description of the demographic characteristics is provided in Supplemental [Supplementary-material supplementary-material-1]. As shown in Table 1, genotype distributions of *miR-34b/c* rs4938723 T>C were compared between all cases and controls. The genotype for *miR-34b/c* rs4938723 T>C was in agreement with the HWE (HWE = 0.784) in the controls. Statistical analysis indicated that rs4938723 C variant allele was associated with decreased neuroblastoma risk (TC vs. TT: adjusted OR = 0.46, 95%CI = 0.30‐0.71; additive model: adjusted OR = 0.64, 95%CI = 0.47‐0.88; TC/CC vs. TT: adjusted OR = 0.49, 95%CI = 0.33‐0.73).

### 3.2. Stratification Analysis

We further demonstrated whether the association between rs4938723 T>C genotype and neuroblastoma risk was modified by age, gender, tumor sites, and INSS stages (Table 2). We observed a significantly decreased risk of neuroblastoma for carriers of TC/CC genotype comparing with carriers of TT genotype in the subgroups of age ≤ 18 months (adjusted OR = 0.35, 95%CI = 0.18‐0.67), age > 18 months (adjusted OR = 0.55, 95%CI = 0.32‐0.94), females (adjusted OR = 0.45, 95%CI = 0.24‐0.82), and males (adjusted OR = 0.52, 95%CI = 0.29‐0.90). Regarding sites of tumor origin, carriers of TC/CC genotype were less likely to have tumors in retroperitoneal (adjusted OR = 0.35, 95%CI = 0.20‐0.60) and other sites (adjusted OR = 0.32, 95%CI = 0.11‐0.94). We also found that the decreased risk of neuroblastoma associated with rs4938723 TC/CC genotypes was more pronounced among clinical stages II (adjusted OR = 0.35, 95%CI = 0.14‐0.89), III (adjusted OR = 0.45, 95%CI = 0.25‐0.83), IV (adjusted OR = 0.26, 95%CI = 0.12‐0.60), and III+IV (adjusted OR = 0.38, 95%CI = 0.23‐0.63).

## 4. Discussion

In our present study, by examining the relationship between *miR-34b/c* rs4938723 T>C and neuroblastoma susceptibility, we identified *miR-34b/c* rs4938723 C allele to be significantly associated with decreased neuroblastoma susceptibility in Hunan children. Our finding, for the first time, implies that *miR-34b/c* rs4938723 T>C protects Chinese children from neuroblastoma risk in Hunan subjects.

*miR-34b* and *miR-34c* are submembers of the *miR-34* family which share a common primary transcript (*pri-miR-34b/c*) [45]. The *miR-34b/c* is located in human chromosome 11 [46, 47]. The biological role of *miR-34b/c* has been well documented in several types of cancers. Majid et al. [48] found that *miR-34b* inhibits prostate cancer through demethylation, active chromatin modifications, and AKT pathways. It is documented that *miR-34b/c* can target *TP53* and cooperate to suppress cell proliferation and adhesion-independent growth [49]. Findings from Wong et al. [50] offer a new insight into the tumor suppressor role of *miR-34b/c* in myeloma. However, there still lacks research regarding the role of *miR-34b/c* in neuroblastoma. The rs4938723 T>C polymorphism, locates within the CpG island of *pri-miR-34b/c*, was intensively investigated. In a study conducted in a Chinese population by Liu et al. [51], they found that CC and TC+CC genotypes of *pri-miR-34b/c* rs4938723 contribute to a higher susceptibility of hepatocellular carcinoma when compared with the TT genotype, respectively. Hashemi et al. [52] found that *miR-34b/c* rs4938723 C allele was correlated with a decreased risk of acute lymphoblastic leukemia, in a sample of an Iranian population with 110 children with acute lymphoblastic leukemia and 120 healthy children. However, Zhu et al. [53] failed to obtain a relationship between *miR-34b/c* rs4938723 and esophageal squamous cell carcinoma risk, in 248 Kazakh patients with esophageal squamous cell carcinoma and 300 frequency-matched control subjects. Polymorphisms may exert distinct genetic effects on the susceptibility of cancer, depending on different cancer types, ethnicities, and regions.

In 2017, we performed a first case-control study regarding *miR-34b/c* rs4938723 T>C and neuroblastoma susceptibility in Chinese children, including 393 cases and 812 controls [37]. We firstly provided an evidence that *miR-34b/c* rs4938723 T>C displayed a protective role from neuroblastoma. However, such evidence needs further validation. Herein, we further verified the protective role of *miR-34b/c* rs4938723 T>C in neuroblastoma risk in another sample of Chinese. Such protective role could also be seen in other cancer types, such as colorectal cancer [34], gastric cancer [54], and esophageal cancer [55]. Other genetic, environmental factors, and gene-environment interaction may cooperatively determine the protective role of *miR-34b/c* rs4938723 T>C in neuroblastoma risk [56, 57].

Strengths of the current study also accompany some limitations. First, statistic power may be compromised as the sample size is not large enough. Second, as a hospital-based case-control study, inclusion of the nonrepresentative subjects in this study may result in inherent selection bias. Third, conclusions obtained here lack generalizability as subjects are all genetic Chinese descent. Therefore, cautions should be taken if the current conclusion is extrapolated to other populations. Fourth, the selected SNP was based on prior knowledge of potentially functional SNPs. Other important tagging SNPs within the *miR-34b/c* gene may be omitted. Last, environment factors and gene-environment interactions could not be assessed in the current study, with the absence of environmental data.

## 5. Conclusions

In conclusion, here, we provided the possibility of *miR-34b/c* rs4938723 T>C in predicting neuroblastoma risk. Our study serves as a basis for future replication studies in independent populations or for functional studies of *miR-34b/c* rs4938723 T>C in neuroblastoma risk.

## Figures and Tables

**Table 1:** *miR34b/c* rs4938723 T>C polymorphism and neuroblastoma susceptibility.

Genotype	Cases (*N* = 162)	Controls (*N* = 270)	*P* ^a^	Crude OR (95% CI)	*P*	Adjusted OR (95% CI)^b^	*P* ^b^
rs4938723 T>C (HWE = 0.784)							
TT	100 (61.73)	117 (43.33)		1.00		1.00	
TC	47 (29.01)	123 (45.56)		**0.45 (0.29-0.69)**	**0.0002**	**0.46 (0.30-0.71)**	**0.0004**
CC	15 (9.26)	30 (11.11)		0.59 (0.30-1.15)	0.119	0.62 (0.32-1.23)	0.175
Additive			0.0008	**0.62 (0.46-0.85)**	**0.0026**	**0.64 (0.47-0.88)**	**0.005**
Dominant	62 (38.27)	153 (56.67)	0.0002	**0.47 (0.32-0.71)**	**0.0002**	**0.49 (0.33-0.73)**	**0.0005**
Recessive	147 (90.74)	240 (88.89)	0.542	0.82 (0.43-1.57)	0.542	0.86 (0.45-1.67)	0.665

OR: odds ratio; CI: confidence interval.^a^*χ*^2^ test for genotype distributions between neuroblastoma cases and cancer-free controls.^b^Adjusted for age and gender.

**Table 2:** Association between *miR34b/c* rs4938723 T>C polymorphism and clinical parameters.

Variables	Cases/controls		OR (95% CI)	*P*	AOR (95% CI)^a^	*P* ^a^
	TT	TC/CC				
	No. (%)	No. (%)				
Age (month)						
≤18	42/36 (25.9)/(13.3)	27/66 (16.7)/(24.4)	**0.35 (0.19-0.66)**	**0.001**	**0.35 (0.18-0.67)**	**0.002**
>18	58/81 (35.8)/(30.0)	35/87 (21.6)/(32.2)	**0.56 (0.34-0.94)**	**0.029**	**0.55 (0.32-0.94)**	**0.027**
Gender						
Females	49/52 (30.2)/(19.2)	30/77 (18.5)/(28.5)	**0.41 (0.23-0.74)**	**0.003**	**0.45 (0.24-0.82)**	**0.010**
Males	51/65 (31.5)/(24.1)	32/76 (19.7)/(28.1)	**0.54 (0.31-0.93)**	**0.027**	**0.52 (0.29-0.90)**	**0.020**
Sites of origin						
Adrenal gland	17/117 (10.5)/(43.3)	14/153 (8.64)/(56.7)	0.63 (0.30-1.33)	0.225	0.65 (0.31-1.38)	0.265
Retroperitoneal	55/117 (33.9)/(43.3)	23/153 (14.2)/(56.7)	**0.32 (0.19-0.55)**	**<0.0001**	**0.35 (0.20-0.60)**	**0.0002**
Mediastinum	16/117 (9.87)/(43.3)	20/153 (12.3)/(56.7)	0.96 (0.48-1.93)	0.900	0.96 (0.48-1.95)	0.917
Others	12/117 (7.41)/(43.3)	5/153 (3.08)/(56.7)	**0.32 (0.11-0.93)**	**0.036**	**0.32 (0.11-0.94)**	**0.039**
Clinical stages						
I	23/117 (14.2)/(43.3)	25/153 (15.4)/(56.7)	0.83 (0.45-1.54)	0.556	0.86 (0.46-1.61)	0.645
II	15/117 (9.26)/(43.3)	7/153 (4.32)/(56.7)	**0.36 (0.14-0.90)**	**0.030**	**0.35 (0.14-0.89)**	**0.027**
III	34/117 (21.0)/(43.3)	20/153 (12.3)/(56.7)	**0.45 (0.25-0.82)**	**0.009**	**0.45 (0.25-0.83)**	**0.010**
IV	28/117 (17.3)/(43.3)	9/153 (5.55)/(56.7)	**0.25 (0.11-0.54)**	**0.0005**	**0.26 (0.12-0.60)**	**0.001**
4s	0/117 (0.00)/(43.3)	1/153 (0.006)/(56.7)	—	—	—	—
I+II+4s	38/117 (23.4)/(43.3)	32/153 (19.7)/(56.7)	0.64 (0.38-1.09)	0.103	0.65 (0.38-1.10)	0.110
III+IV	62/117 (38.3)/(43.3)	29/153 (17.9)/(56.7)	**0.36 (0.22-0.59)**	**<0.0001**	**0.38 (0.23-0.63)**	**0.0002**

OR: odds ratio; CI: confidence interval; AOR: adjusted odds ratio.^a^Adjusted for age and gender, omitting the corresponding stratify factor.

## Data Availability

The data used to support the findings of this study are available from the corresponding authors upon request.

## Abbreviations

miRNA: MicroRNA
SNP: Single nucleotide polymorphism
HWE: Hardy-Weinberg equilibrium
OR: Odds ratio
CI: Confidence interval.

## Supplementary Materials

Supplementary Materials Supplemental Table 1: frequency distribution of selected characteristics in neuroblastoma cases and cancer-free controls for Hunan children.
