# Supplementary material for: miR-34b/c rs4938723 T>C Decreases Neuroblastoma Risk: A Replication Study in the Hunan Children
Source: Dis Markers. 2019 Sep 10;2019:6514608. doi: 10.1155/2019/6514608 (PMC6754906; doi:10.1155/2019/6514608)
Supplement: Supplementary Materials — Supplemental Table 1: frequency distribution of selected characteristics in neuroblastoma cases and cancer-free controls for Hunan children. [file 6514608.f1.doc]

| **Supplemental Table 1**. Frequency distribution of selected characteristics in neuroblastoma cases and cancer-free controls for Hunan children | | | | | |
| --- | --- | --- | --- | --- | --- |
| Variables | Cases (n=162) | | Controls (n=270) | | *P* ^a^ |
|  | No. | % | No. | % |  |
| Age range, month | 0.033-130.00 | | 0.033-101.00 | | 0.322 |
| Mean ± SD | 34.56±30.30 | | 27.81±19.83 | |  |
| ≤18 | 69 | 42.59 | 102 | 37.78 |  |
| >18 | 93 | 57.41 | 168 | 62.22 |  |
| Gender |  |  |  |  | 0.842 |
| Female | 79 | 48.77 | 129 | 47.78 |  |
| Male | 83 | 51.23 | 141 | 52.22 |  |
| INSS stages |  |  |  |  |  |
| I | 48 | 29.63 | / | / |  |
| II | 22 | 13.58 | / | / |  |
| III | 54 | 33.33 | / | / |  |
| IV | 37 | 22.84 | / | / |  |
| 4s | 1 | 0.62 | / | / |  |
| NA | / | / | / | / |  |
| Sites of origin |  |  |  |  |  |
| Adrenal gland | 31 | 19.14 | / | / |  |
| Retroperitoneal region | 78 | 48.15 | / | / |  |
| Mediastinum | 36 | 22.22 | / | / |  |
| Other region | 17 | 10.49 | / | / |  |
| NA | / | / | / | / |  |
| SD, standard deviation; NA, not available.  ^a^ Two-sided χ^2^ test for distributions between neuroblastoma cases and cancer-free controls. | | | | | |
